# Remote Ischemic Preconditioning Protects against Liver Ischemia-Reperfusion Injury via Heme Oxygenase-1-Induced Autophagy

**DOI:** 10.1371/journal.pone.0098834

**Published:** 2014-06-10

**Authors:** Yun Wang, Jian Shen, Xuanxuan Xiong, Yonghua Xu, Hai Zhang, Changjun Huang, Yuan Tian, Chengyu Jiao, Xuehao Wang, Xiangcheng Li

**Affiliations:** 1 Key Laboratory of Living Donor Liver Transplantation, Ministry of Public Health, Department of Liver Transplantation Center, The First Affiliated Hospital of Nanjing Medical University, Nanjing, Jiangsu Province, China; 2 Department of Oncological Surgery 2, Xuzhou City Central Hospital, The Affiliated Hospital of the Southeast University Medical School (Xuzhou), The Tumor Research Institute of the Southeast University (Xuzhou), Xuzhou, Jiangsu Province, China; 3 Department of General Surgery, The Second Affiliated Hospital of Nanjing Medical University, Nanjing, Jiangsu Province, China; 4 Department of Gastroenterology II, Xuzhou City Central Hospital, The Affiliated Hospital of the Southeast University Medical School (Xuzhou), Xuzhou, Jiangsu Province, China; 5 Department of General Surgery, Yancheng First People's Hospital, Yancheng, Jiangsu Province, China; 6 Department of General Surgery, The First Affiliated Hospital of Jiangsu University, Zhenjiang, Jiangsu Province, China; University Hospital Heidelberg, Germany

## Abstract

**Background:**

Growing evidence has linked autophagy to a protective role of preconditioning in liver ischemia/reperfusion (IR). Heme oxygenase-1 (HO-1) is essential in limiting inflammation and preventing the apoptotic response to IR. We previously demonstrated that HO-1 is up-regulated in liver graft after remote ischemic preconditioning (RIPC). The aim of this study was to confirm that RIPC protects against IR via HO-1-mediated autophagy.

**Methods:**

RIPC was performed with regional ischemia of limbs before liver ischemia, and HO-1 activity was inhibited pre-operation. Autophagy was assessed by the expression of light chain 3-II (LC3-II). The HO-1/extracellular signal-related kinase (ERK)/p38/mitogen-activated protein kinase (MAPK) pathway was detected in an autophagy model and mineral oil-induced IR in vitro.

**Results:**

In liver IR, the expression of LC3-II peaked 12–24 h after IR, and the ultrastructure revealed abundant autophagosomes in hepatocytes after IR. Autophagy was inhibited when HO-1 was inactivated, which we believe resulted in the aggravation of liver IR injury (IRI) in vivo. Hemin-induced autophagy also protected rat hepatocytes from IRI in vitro, which was abrogated by HO-1 siRNA. Phosphorylation of p38-MAPK and ERK1/2 was up-regulated in hemin-pretreated liver cells and down-regulated after treatment with HO-1 siRNA.

**Conclusions:**

RIPC may protect the liver from IRI by induction of HO-1/p38-MAPK-dependent autophagy.

## Introduction

Liver ischemia/reperfusion (IR) injury (IRI) is a phenomenon in which cellular damage caused by hypoxia is accentuated following return of blood flow and restoration of oxygen delivery. This remains an important clinical problem during shock, hepatic resection, and liver transplantation. Surgical, pharmacologic strategies, and gene therapy are the major methods used to alleviated liver IRI [1], [2]. The first work on remote ischemic preconditioning (RIPC), described by Przyklenk, et al in 1993, showed that brief occlusion of the circumflex artery protects myocardium from subsequent continuous IRI. This proved to be a novel and simple way to protect the liver without direct stress [3]. RIPC involves brief periods of ischemia followed by reperfusion of one organ or tissue that subsequently affords protection to a remote organ or tissue suffering from a prolonged ischemic injury [4].

The heme oxygenase (HO) system is part of a vital cell-signaling pathway that occurs in response to cellular injury or stress. Overexpression of HO-1 may protect the liver from IRI via anti-inflammatory and anti-apoptotic effects. Pharmacologic-induced upregulation of HO-1 is protective of cells and organs, whereas HO-1 inhibition may abolish the effect and aggravate the damage [5]–[7]. Studies have shown that overexpression of HO-1 induced by transient limb ischemia may play a protective role in hepatic IRI in rats [8]. Our previous work revealed that RIPC may induce HO-1 to protect the liver from IRI in a small-for-size liver transplantation model. However, the molecular mechanism of the protective role of HO-1 induced by RIPC remains unclear.

Autophagy (Greek for “self-eating”) is a general term for processes in which cytoplasmic materials, including organelles, are wrapped up by a double membrane vesicle, termed an autophagosome, for degradation. It is primarily categorized as a process incited by nutrient starvation. Growing evidence has revealed that autophagy plays a protective role in liver IRI, partly by consuming damaged and dysfunctional mitochondria to prevent the release of cytochrome C and mitochondrial death signaling, and potentially contributes to the regulation of oxygen consumption. A study showed that decreased autophagic levels may lead to the increased sensitivity of aged livers to IRI both in vitro and in vivo. Furthermore, it may yield a novel strategy to ameliorate the effects of liver IR via inducing autophagy [9]. Autophagy is an adaptive response in liver IR models to limit cellular death and organ damage. Additionally, HO-1 has been recognized as a protein that is essential to limiting inflammation and preventing cell death or apoptosis, however the mechanisms, including a link to autophagy, are not well defined. Given this, we hypothesized that autophagy is regulated by HO-1, which is induced by RIPC to protect the liver from IR injury.

## Materials and Methods

### Animals and ethics statement

Male ICR (Institute of Cancer Research) wild-type mice (weighing 28–32 g), purchased from the animal center of Nanjing Medical University, were housed under specific pathogen-free conditions. All animals received humane care according to the Common standards and were kept under constant environmental conditions. Animal experiments were conducted in accordance with the guidelines approved by the China Association of Laboratory Animal Care. The protocol was approved by the Committee on the Ethics of Animal Experiments of the Nanjing Medical University (Permit Number: NJMU-AEARIA-4001-20120401). All surgeries were performed under sodium pentobarbital anesthesia, and all efforts were made to minimize suffering.

### Animal model and treatment

Warm liver IRI was carried out as described by Shan Zeng et al. Briefly, mice were anesthetized with sodium chloralic hydras (30 mg/kg, intraperitoneal), and a midline incision was performed. The blood supply for the median and lateral left lobes were interrupted by a vascular microclamp for 45 min. Reperfusion was initiated by removal of the clamp [10]. Sham controls only received 4 ml/kg of 0.9% NaCl and the operation but no vascular occlusion. To inhibit the activity of HO-1, mice were treated with Zinc protoporphyrin (Znpp, 10 µmol/kg, Sigma-Aldrich, St. Louis, MO) at 16 h and 3 h preoperation, as described previously [11].

For RIPC, a longitudinal skin incision was performed from the umbilical midline level to the right knee. The femoral vascular bundle (femoral artery and vein) was isolated from the surrounding muscles and clamped with a vascular microclamp. Six cycles of 4 min of limb ischemia followed by 4 min of reperfusion were subsequently performed [12].

### Experimental groups and sample collection

One hundred and fifteen mice were randomly divided into five different treatment groups as follows: sham group (5 mice); IR group (35 mice with 5 mice in each of 7 subgroups divided by reperfusion times of 0.5, 2, 6, 12, 24, 48, and 72 h); RIPC+IR group (5 mice underwent RIPC only and 20 mice underwent RIPC before IR with 5 mice in each of 4 subgroups divided by reperfusion times of 2, 6, 12, and 24 h); Znpp+IR group (5 mice underwent Znpp only and 20 mice underwent Znpp administration before IR with 5 mice in each of 4 subgroups divided by reperfusion times of 2, 6, 12, and 24 h); and Znpp+RIPC+IR group (5 mice underwent Znpp administration following RIPC and 20 mice underwent Znpp administration following RIPC before IR with 5 mice in each of 4 subgroups divided by reperfusion times of 2, 6, 12, and 24 h). Prior to the operation, 4 ml/kg of 0.9% NaCl was administrated in the sham, IR and RIPC+IR groups. Mice were sacrificed at different times after reperfusion. Blood samples were stored at 4°C overnight, centrifuged for 15 min at 3,000 rpm, and supernatants were collected and underwent serological testing. Liver parenchymas were divided into two parts: one was snap-frozen in liquid nitrogen and the other tissue specimens were paraffin-embedded after formalin fixation for hemotoxylin and eosin (H&E) staining and immunohistochemistry. Blood samples collected at 2, 6, 12 and 24 h after reperfusion were used to measure the alanine aminotransferase (ALT) and aspartate aminotransferase (AST) levels with a standard automatic biochemistry analyzer. The cultured supernatants of 2, 6, 12, and 24 h were transferred to new tubes in the medium exchange, and were used to measure ALT and AST levels.

### Histology

Standard H&E staining was performed on representative 3 µm sections of each case for general histopathologic evaluation of IRI according to Suzuki's classification, in which sinusoidal congestion, hepatocyte necrosis, and ballooning degeneration are graded from 0 to 4 (Suzuki et al, 1993) [13].

### Small interfering RNA (siRNA) transfection

Small interfering RNAs (siRNAs) against HO-1 and a nonspecific scrambled siRNA were purchased from Ambion (Austin, TX). All siRNAs were synthesized by Qiagen (Chatsworth, CA). AML12 (alpha mouse liver 12) cells were cultured in 6-well plates overnight to ∼80–90% confluence. Lipofectamine 2000 (Invitrogen, Rockville, MD) was mixed into Dulbecco's modified Eagle's medium (DMEM) without 10% heat-inactivated fetal bovine serum (FBS) containing siRNA1, siRNA2, or scrambled RNA, respectively, according to the manufacturer's protocol. Additionally, mock controls were transfected with Lipofectamine 2000 (Invitrogen) alone, incubated at room temperature for 20 min, and distributed into duplicate wells. Subsequently, transfection was performed at 37°C in 5% CO_2_. The medium was replaced with DMEM containing 10% heat-inactivated FBS for 4–6 h. After 2 h, cells were treated as indicated below.

### Cell culture and treatment

The AML12 (alpha mouse liver 12) cell line were purchased from the cell bank at Shanghai Institutes for Biological Sciences, Chinese Academy of Sciences, Shanghai. Cells were cultured in DMEM supplemented with 10% heat-inactivated FBS and 100 U/ml penicillin, 100 U/ml streptomycin (Life Technologies, Carlsbad, CA). Cells were plated at density of 2×10^6^ cells/mL in 6-well plates for cell viability analysis, disposing, and western blot assays.

Cells were divided into nine groups according to the different processing methods: (1) control group, cells were cultured in DMEM without any dispose; (2) IR group, cells were immersed in mineral oil (1 ml/well, Sigma-Aldrich) for 1 h to simulate ischemia [14] and washed twice with PBS before being cultured in DMEM for 2, 6, 12, or 24 h to simulated reperfusion; (3) HIR (hemin pretreatment before IR simulation) group, cells were pretreated with DMEM in the presence of 20 µmol/L hemin (Sigma-Aldrich) for 12 h [15], washed twice with PBS, immersed in mineral oil, and cultured in DMEM for 2, 6, 12, or 24 h to simulate reperfusion; (4) SSIR (scrambled siRNA+S+IR) group, cells transfected with scrambled siRNA were immersed in Hank's balanced salt solution (HBSS) containing Ca^2+^ and Mg^2+^ in 10 mM HEPES (1 mL/well, Sigma-Aldrich) for 0.5 h to induce autophagy [16] before growth in mineral oil and DMEM for 2, 6, 12, or 24 h to simulate IR; (5) HSIR (HO-1 siRNA+S+IR) group, cells were transfected with HO-1 siRNA1 or siRNA2 for 2 h as described above, and then the cells were immersed in HBSS containing Ca^2+^ and Mg^2+^ in 10 mM HEPES for 0.5 h to induce autophagy before growth in mineral oil and DMEM for 2, 6, 12, or 24 h to simulate IR; (6) EI (ERK inhibitor PD98059) group, PD98059 (20 µmol; Cell Signaling Technology Inc, Danvers, MA) was used to culture AML12 cells for 1 h; (7) EIIR (PD98059 + IR) group, PD98059 was used to culture AML12 cells for 1 h [17], and the cells were then immersed in mineral oil and cultured in DMEM for 2, 6, 12, or 24 h to simulate reperfusion; (8) PI (p38 MAPK inhibitor SB203580) group, SB203580 (20 µmol; Calbiochem, San Diego, CA) was used to culture AML12 cells for 1 h; (9) PIIR (SB203580 + IR) group, SB203580 was used to culture AML12 cells for 1 h [18], and then the cells were immersed in mineral oil and cultured in DMEM for 2, 6, 12, or 24 h to simulate reperfusion.

### Reverse transcription-quantitative real-time polymerase chain reaction (RT-qPCR)

Total cellular RNA was isolated and purified using the Trizol reagent. cDNA was synthesized using the PrimeScript RT Master Mix in a 10-µL reaction. According to the SYBR Premix Ex Taq real-time PCR kit, we used cDNA (2 µL) as a template in a 20-µL reaction. Primers were synthesized by Invitrogen (Shanghai, China). The primers for HO-1 were as follows: 5′-GTCAAGCACAGGGTGACAGA-3′ (sense) and 5′-CTGCAGCTCCTCAAACAGC-3′ (antisense). β-actin was used as the reference gene in our experiment; the primers for β-actin were as follows: 5′-TCACCCACACTGTGCCCATCTACGA-3′ (sense) and 5′-CAGCGGAACCGCTCATTGCCAATGG-3′ (antisense). For RT-PCR, we used the following cycles: 95°C for 30 sec, 40 cycles of 95°C for 5 sec, and 60°C for 31 sec, and the dissociation stage was 95°C for 15 sec, 60°C for 1 min, and 95°C for 15 sec.

### Western blot analysis

Frozen liver tissues and cultured cells were homogenized in RIPA lysis buffer (Biyotime, Haimen, China) in the presence of 1% (v/w) protease inhibitor cocktail (Pierce Biotechnology, Rockford, IL). The mixture was placed on a shaker at 4°C for 1 h and insoluble matter was removed by centrifugation at 40000× *g* at 4°C for 1 h. Protein concentration was determined by the Bradford method using bovine serum albumin (BSA) as the standard. Proteins were resolved on sodium dodecyl sulfate polyacrylamide gel electrophoresis gels and transferred onto polyvinylidene difluoride membranes (Millipore, Bedford, MA). The membrane was incubated overnight with primary antibodies to LC3-II, p38 mitogen activated protein kinase (MAPK), p-ERK(Novus Biologicals, Littleton, CO), or HO-1(Abcam, Cambridge, UK). β-actin expression was used as a loading control(Abcam). Membranes were incubated with horseradish peroxidase (HRP)-conjugated secondary antibody (1∶4000; Beijing ZhongShan Biotechnology, Beijing, China) for 1 h and visualized using an enhance chemiluminesence detection kit following the manufacturer's instructions (Pierce-Thermo Scientific, Rockford, IL). All proteins were detected in more than three independent experiments, and for each protein, the gray value was measured by Image-Pro Plus 6.0 in order to conduct statistical analyses.

### Electron microscopic analysis

Liver segments were harvested as soon as blood samples were collected. They were cut into 1 mm^3^ in size for fixation by 2% glutaraldehyde in 0.1 mol/L PBS (PH = 7.4). AML12 cells were grown on six-well plates and harvested by the addition of pancreatic enzymes before fixation in 2% glutaraldehyde in 0.1 mol/L PBS (pH = 7.4). For ultrastructural examination, tissues and cells that had been prepared were postfixed with 2% OsO_4_ and embedded in Araldite. Ultrathin sections were stained with uranyl acetate and lead citrate, and inspected using an electron microscope (JEM.1010; JEOL, Tokyo, Japan).

### Immunohistochemistry and immunofluorescence

Serial 4-µm-thick sections of each specimen were deparaffined and hydrated before antigen retrieval by 10 mM citric acid buffer (pH 7.0). After eliminating endogenous peroxidase activity, the specimens were blocked with blocking serum and incubated at 4°C overnight with anti-LC3-II primary antibody (1∶200). Sections were incubated with HRP-conjugated secondary antibody for 1 h the next day and visualized by diaminobenzidine. Images were obtained by bright-field microscopy (Axioskop 2 plus, ZEISS, Germany). Negative controls were incubated with a solution devoid of any primary antibody.

AML12 cells were prepared on six-well plates that were first fixed on coverslips with 4% paraformaldehyde for 20 min. After rinsing with PBS 4×10 min, cells were blocked in 10% BSA for 1 h and incubated in primary antibody (anti- LC3II in 10% BSA 1∶100) overnight. Cells were cultured with secondary antibody (1∶100) for 1 h at 37°C the next day and nuclei were stained with 4′, 6-diamidino-2-phenylindole (DAPI) before observation.

Five visual fields were randomly chosen, and semi-quantitative analyses of the immunohistochemistry and immunofluorescence results were performed using Image-Pro Plus 6.0. We then constructed histograms according to the photometric values.

### Apoptosis analysis

Apoptosis in AML12 cells was quantified by double staining with annexin V-fluorescein isothiocyanate (FITC) and propidium iodide (PI). Cells (5×10^5^) were collected and washed twice with ice-cold PBS and resuspended in 500 µl of binding buffer. Subsequently, 5 µl of Annexin V-FITC and 5 µl of PI were added, mixtures were incubated for 5–15 min in the dark at 4°C. After 1 h of incubation, samples were analyzed by FACS Calibur flow cytometer (BD, San Jose, CA, USA) using Cell Quest software (BD).

### Statistical analysis

Data are presented as mean ± standard deviation. Analysis of variance, q test and Student's t tests were performed for the parameters, enzyme levels (ALT and AST), gray value of protein, photometric values and Suzuki's classification. P<0.05 was considered statistically significant. SPSS11.0 software was used in all statistical analyses.

## Results

### Autophagy was induced in mouse liver ischemia reperfusion injury

**Figure 1:**
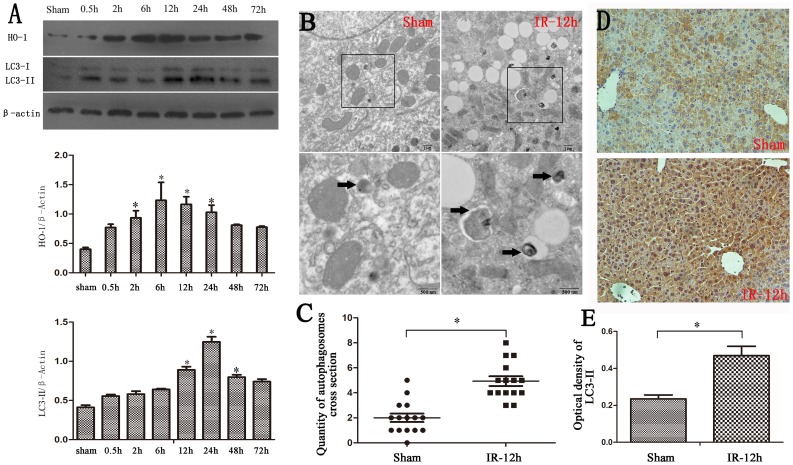
Hepatic inflow occlusion and reflow increased hepatic HO-1 and autophagic signaling. (A) Western blotting for HO-1 and LC3-II in liver lysates from sham-operated or IR-treated mice shows increased HO-1 and LC3-II in the IR group, and the expression of HO-1, LC3-II peaked at 6–12 h and 12–24 h, respectively(*P<0.05 compared with the sham group). (B) Transmission electron microscopy of a 12-h liver sample after reperfusion revealed increased autophagosome formation (black arrows). (C) The data were quantified by counting the number of autophagosomes per cross-sectioned cell (*P<0.05 compared with sham group, n = 15). (D) The intensity of immunostaining of LC3-II (yellow) significantly increased after IRI compared with the sham group (*P<0.05 compared with the sham group). (E) The optical density of LC3-II immunostaining in the 12-h IR group was higher than in the sham group (*P<0.05 compared with the sham group).

ICR mice were randomized to a sham or liver IR operation. As shown in Fig. 1, the expression of LC3-II was up-regulated during liver IR, and peaked at 12–24 h. The expression of HO-1 was up-regulated at the same time and peaked at 6–12 h (Fig. 1A). Consistently, the ultrastructure of the liver after IR revealed that there were abundant autophagosomes, which were rarely seen in the sham group by transmission electron microscopy (TEM) (Fig. 1B). The intensity of LC3-II immunostaining also significantly increased after IRI compared with the sham group (Fig. 1D).

### RIPC induced HO-1 expression and autophagy and alleviated liver ischemia-reperfusion injury

**Figure 2:**
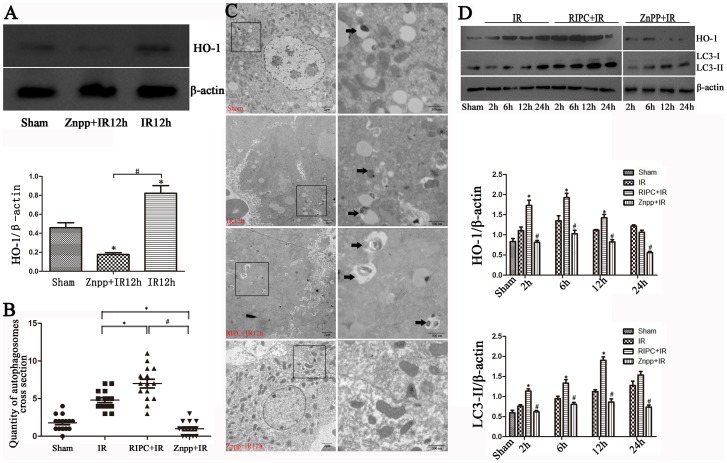
Remote ischemic preconditioning induced HO-1 to increase autophagy, but the inhibition of HO-1 by Znpp decreased autophagy. (A) HO-1 was downregulated in the 12-h Znpp+IR group compared with the sham and 12-h IR groups (*P<0.05 compared with the sham group; ^#^P<0.05 compared with the IR group). (B) The data were quantified by counting the number of autophagosomes per cross-sectioned cell (*P<0.05 compared with the IR group; ^#^P<0.05 compared with the RIPC+IR group, n = 15). (C) IR treatment increased autophagy compared to sham as observed by transmission electron microscopy and increased autophagosome formation in the RIPC+IR group, whereas autophagosomes were rare in the Znpp+IR group (black arrows). (D) Increased HO-1 and LC3-II staining was observed in the RIPC+IR group compared with the IR group, and LC3-II staining was decreased after inhibition of HO-1 (*P<0.05 compared with the IR group at the corresponding time point; ^#^P<0.05 compared with the RIPC+IR group at the corresponding time point).

**Figure 3:**
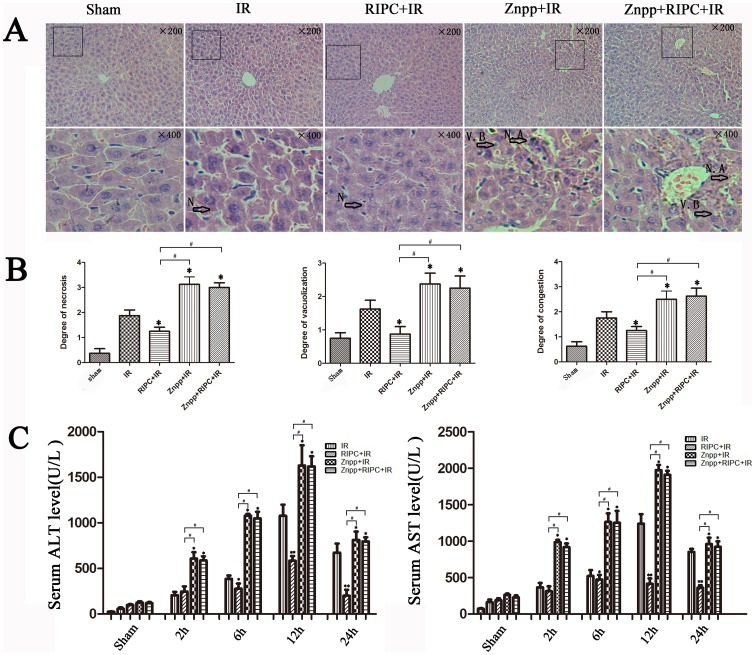
Increased HO-1 and autophagy alleviated liver damage. (A) IR treatment increases the histopathologic changes of liver. Extensive areas of hepatocyte necrosis (N), vacuolation (V) and sinusoidal congestion were observed in the IR group. The Znpp+IR and Znpp+RIPC+IR group showed markedly higher hepatocyte necrosis accumulation (N. A) and vacuo(V), whereas the RIPC+IR group still showed large areas of normal liver architecture, similar to the sham group mice (original magnification 200×; insets 400×). (B) The mean injury score of the RIPC+IR group was significantly lower than the IR group according to Suzuki's histologic classification. The mean injury scores in the Znpp+IR and Znpp+RIPC+IR groups were significantly higher than in the IR and RIPC+IR groups (*P<0.05 compared with the IR group; ^#^P<0.05 compared with the RIPC+IR group). (C) IR treatment increased serum aminotransferase levels in mice compared with controls. Compared with the IR group, serum aminotransferase levels were lower in the RIPC+IR group. The mean serum levels of ALT and AST were highest in the Znpp+IR and Znpp+RIPC+IR groups. There were no significant differences in aminotransferase levels in the Znpp+IR and Znpp+RIPC+IR groups (*P<0.05 compared with the IR group; ^#^P<0.05 compared with the RIPC+IR group).

We performed a similar experiment with the IR mouse model that also underwent RIPC. We found increases in HO-1 and LC3-II expression levels in the RIPC+IR group compared with the sham and IR groups (Fig. 2D). The ultrastructure of the liver also showed that there were more autophagosomes in the RIPC+IR group than in the IR group (Fig. 2C). We recapitulated previous results indicating that IR injury may be alleviated by hindlimb RIPC [12], which was confirmed by the result of H&E staining (Fig. 3A) and liver function tests (Fig. 3C). The mean serum levels of ALT and AST peaked 12 h after reperfusion in all groups, and were significantly higher in the IR and RIPC+IR groups compared with the sham (Fig. 3C). However, the ALT and AST levels were lower in the RIPC+IR group than the IR group (12 h and 24 h, P<0.05).

After reperfusion, livers showed extensive areas of hepatocyte necrosis and sinusoidal congestion in the IR group, particularly in the area around the central vein. Histological examination showed that RIPC tended to decrease the incidence of hepatocyte necrosis, ballooning degeneration, and sinusoidal congestion (Fig. 3B). According to Suzuki's histologic classification, the mean injury score in the RIPC+IR group was significantly lower than that in the IR group (P<0.05).

### Znpp decreased autophagy and aggravated liver damage

Cells were pretreated with Znpp before IR, and the subsequent inhibition of HO-1 was detected. As shown in Figure 2A, HO-1 was down-regulated in the Znpp+IR group compared with the sham group. IRI appeared to be aggravated when HO-1 was inhibited by Znpp in the Znpp+IR group. LC3-II staining was decreased by inhibiting of HO-1 and TEM also showed decreased autophagosomes in the Znpp+IR group (Fig. 2C, D), which significantly raised serum ALT and AST levels in the Znpp+IR group (Fig. 3C). There was greater necrosis, ballooning degeneration, and sinusoidal congestion in the Znpp+IR group compared to the other three groups (Fig. 3A and B), and the mean injury score in the Znpp+IR group was significantly higher than that in the IR and RIPC+IR groups according to Suzuki's histologic classification (P<0.05).

### The protective role of autophagy was dependent on HO-1 in vivo

**Figure 4:**
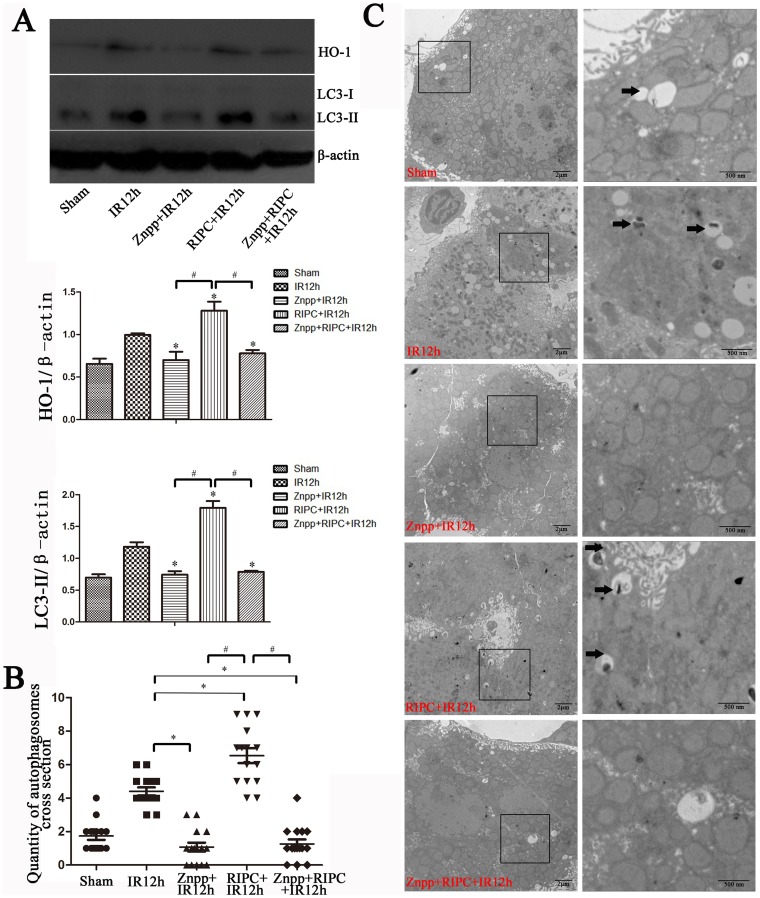
Induction of autophagy was dependent on HO-1 in vivo. (A) Increased HO-1 and LC3-II staining was observed in the RIPC+IR group compared with the Sham, IR, Znpp+IR, and Znpp+RIPC+IR groups, and there were no significant differences in the expression of HO-1 and LC3-II protein between the Znpp+IR and Znpp+RIPC+IR groups (*P<0.05 compared with the IR group; ^#^P<0.05 compared with the RIPC+IR group). (B) The data were quantified by counting the number of autophagosomes per cross-sectioned cell (*P<0.05 compared with the IR group; ^#^P<0.05 compared with the RIPC+IR group, n = 15). (C) IR treatment increased autophagy as observed by transmission electron microscopy compared to the sham group and showed increased autophagosome formation in the RIPC+IR group, whereas autophagosomes were rare in the Znpp+IR and Znpp+RIPC+IR groups.

To study the relationship between HO-1 and autophagy, the Znpp+RIPC+IR group was used. As seen in Figure 3, we found no significant differences in liver function and pathological damage in the Znpp+IR and Znpp+RIPC+IR groups. HO-1 and LC3-II staining in the Znpp+IR group was nearly the same as that in the Znpp+RIPC+IR group, which was confirmed by western blotting (Fig. 4A) and TEM (Fig. 4C). Increases in HO-1 and LC3-II expression levels were found in the RIPC+IR group compared with those in the sham, IR, Znpp+IR, and Znpp+RIPC+IR groups (Fig. 4A and C).

### Inhibition of HO-1 increased biochemical enzyme levels of cultural supernatants and cell death, although it decreased autophagy in vitro

To further elucidate the relationship between HO-1 and autophagy, we established cell models of IR and autophagy in vitro. IR was simulated in vitro by mineral oil, whereas HO-1 was induced by hemin media and inhibited by HO-1 SiRNA as described previously.

**Figure 5:**
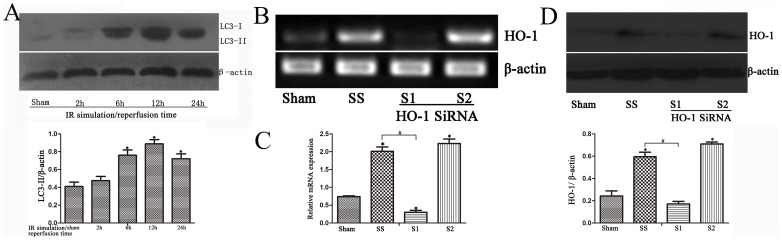
IR simulation in AML12 cells increased LC3-II expression; HO-1 siRNA effectively inhibited HO-1 expression. (A) LC3-II staining increased in the IR cell model and peaked at 12–24 h (*P<0.05 compared with the 0-h group). (B and C) After transfection into AML12 cells, siRNA1 (S1) was shown to significantly inhibit HO-1 mRNA compared with siRNA2 (S2), whereas scrambled siRNA (SS) had no measurable effect on HO-1 expression (*P<0.05 compared with the sham group; ^#^P<0.05 compared with the SS group). (D) Western blotting showed lower expression of HO-1 protein in the S1 group compared with the SS and S2 groups (*P<0.05 compared with the sham group; ^#^P<0.05 compared with the SS group).

We selected two HO-1 siRNAs, siRNA1 (S1) and siRNA2 (S2), as well as a scrambled siRNA (SS). Following transfection into AML12 cells, HO-1 expression was detected by RT-PCR and western blotting. siRNA1 (S1) was shown to inhibit HO-1 significantly, whereas the scrambled siRNA (SS) had no measurable effect on HO-1 expression (Fig. 5B and D). We thus subsequently used siRNA1 in our experiments.

**Figure 6:**
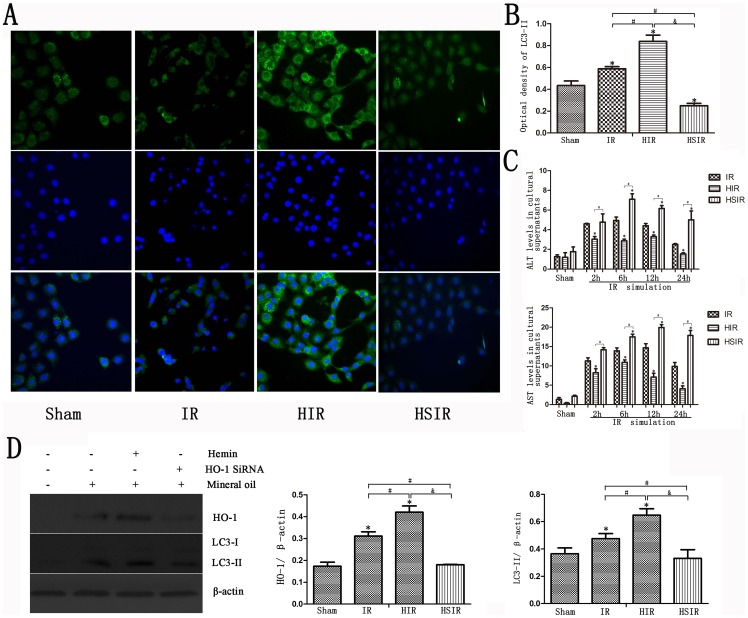
Inhibition of HO-1 increased cell death and decreased autophagy in AML12 cells. (A) Immunofluorescence of AML12 cells demonstrated increased LC3-II staining in the HIR group; however, downregulated HO-1 decreased autophagy. (B) Increased photometric values of protein in the HIR group compared with the IR and HSIR groups are shown, and the lowest photometric values of protein were observed in the HSIR group (*P<0.01 compared with the sham group; ^#^P<0.05 compared with the IR group; ^&^P<0.05 compared with the HIR group). (C) Induction of HO-1 and autophagy decreased biochemical enzyme levels, whereas inhibition of HO-1 and autophagy increased biochemical enzyme levels (*P<0.01 compared with the IR group; ^#^P<0.05 compared with the HIR group). (D) The expression of HO-1 and autophagy proteins was upregulated by hemin, but downregulated by treatment with HO-1 siRNA (*P<0.01 compared with the sham group; ^#^P<0.05 compared with the IR group; ^&^P<0.05 compared with the HIR group).

**Figure 7:**
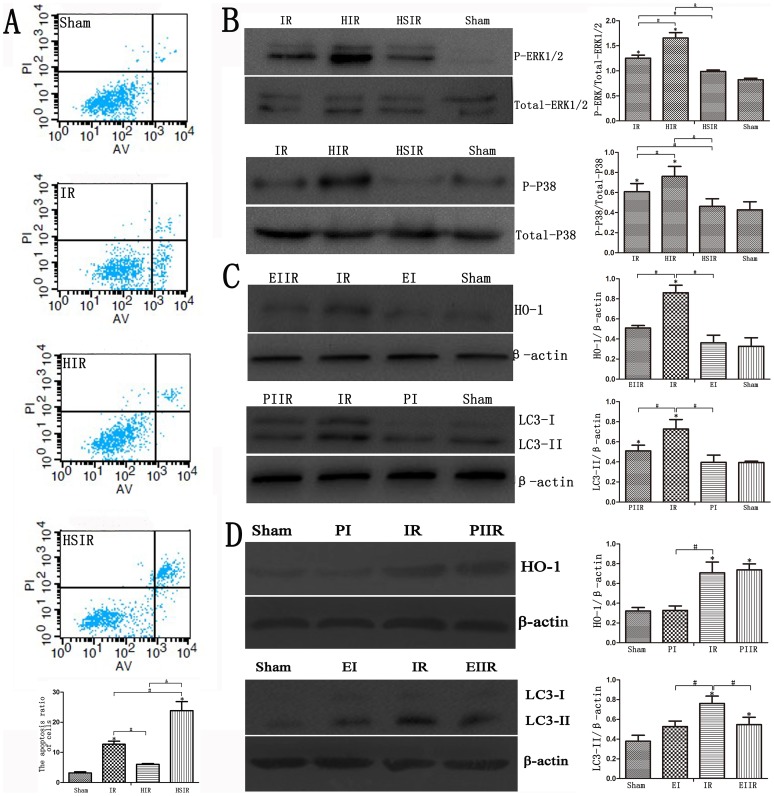
HO-1 induced autophagy through activation of ERK1/2 and p38-MAPK. (A) Apoptotic rates of the sham group and three treated groups are shown. Mineral oil treatment increased the rate of early and late apoptosis, and HO-1 siRNA treatment before IR increased the rate of apoptosis compared with the IR group. The HIR group had lower rates of apoptosis, similar to the sham group (*P<0.01 compared with the sham group; ^#^P<0.05 compared with the IR group; ^&^P<0.05 compared with the HIR group). (B) Hemin treatment before IR increased phosphorylated ERK1/2 staining, as shown in immunoblotting, and HSIR group cells showed decreased phosphorylated ERK1/2 expression. Western blotting for phosphorylated p38-MAPK demonstrated increased phosphorylated p38-MAPK in the HIR group, but decreased signaling in the HSIR group (*P<0.01 compared with the sham group; ^#^P<0.05 compared with the IR group; ^&^P<0.05 compared with the HIR group). (C) HO-1 expression was decreased in the EIIR group compared with the IR group, and LC3-II expression was decreased in the PIIR group compared with the IR group (*P<0.05 compared with the sham group; ^#^P<0.05 compared with the IR group). (D) HO-1 expression was nearly the same as in the PIIR and IR groups, but LC3-II expression was decreased in the EIIR group compared with the IR group (*P<0.05 compared with the sham group; ^#^P<0.05 compared with the IR group).

As shown in Figure 5A, LC3-II staining increased in the IR cell model and peaked at 12–24 h (Fig. 5A). Immunofluorescence showed that increased LC3-II staining was observed in the HIR group, but down-regulated HO-1 decreased autophagy (Fig. 6A). We found increased photometric values of protein in the HIR group compared with the IR and HSIR groups, and the lowest photometric values of protein were found in the HSIR group (Fig. 6B). The expression levels of HO-1 and autophagy proteins were up-regulated using hemin but down-regulated after treatment with HO-1 siRNA (Fig. 6D). This result was verified by statistical analyses of the gray values. Furthermore, induction of HO-1 and autophagy decreased biochemical enzyme levels, whereas inhibition of HO-1 and autophagy increased biochemical enzyme levels (Fig. 6C). Annexin-V/PI staining showed that apoptosis was increased in all of the three treated groups; mineral oil treatment increased the rate of early and late apoptosis, and HO-1 SiRNA treatment before IR increased the apoptosis rate compared with IR group (P<0.05). However, there was no significant differences of apoptosis rates between the control group and the HIR group (P>0.05) (Fig. 7A).

### HO-1-induced autophagy through activation of ERK1/2 and p38-MAPK

To reveal the molecular mechanism of the protective role of HO-1-induced autophagy in IR, we detected the activation of MAPKs, which had been showed to modulate induction of HO-1 and autophagic signaling [14], [31]. Hemin pre-treatment IR enhanced the expression of HO-1 staining and increased extracellular signal-related kinase (ERK)1/2 and p38-MAPK phosphorylation (Fig. 7B). Conversely, down-regulation of HO-1 decreased the phosphorylation of ERK1/2 and p38-MAPK. (Fig. 7B). The statistical analyses of the gray values showed the same result, but there were no statistical differences in the gray values representing ERK1/2 phosphorylation between the HSIR and IR groups (P>0.05).

To further identify the signaling pathways of HO-1 and autophagy, we used the ERK inhibitor PD98059 and the p38 MAPK inhibitor SB203580. We determined whether ERK pathways were involved in the induction of HO-1 expression, and whether p38 MAPK pathways were involved in the induction of autophagy.

As shown in Figure 7C, HO-1 expression was blocked by PD98059, and LC3-II expression was down-regulated by SB203580. Additionally, LC3-II expression was inhibited by PD98059, but HO-1 expression was not influenced by SB203580 (Fig. 7D).

## Discussion

In summary, we found that autophagy in a hepatic inflow occlusion and reflow in a mouse model or in an in vitro mineral oil treatment model is dependent upon HO-1. Induction of HO-1 in the mouse by RIPC or induced HO-1 in cells by hemin increased autophagy and alleviated liver injury. Similarly, inhibition of HO-1 expression by Znpp in vivo, or down-regulation of HO-1 SiRNAs in vitro, may decrease LC3-II protein expression which resulted in hepatic damage or cell death. To further study the relationship between HO-1 and autophagy, the Znpp+RIPC+IR group was used. HO-1 and LC3-II expression was induced in the RIPC+IR group, but when HO-1 expression was inhibited by Znpp in the Znpp+RIPC+IR group, we saw no increase in HO-1 expression by RIPC following induction of LC3-II expression. Hepatic damage was not alleviated in the Znpp+RIPC+IR group compared with the RIPC+IR group. We thus presumed that RIPC could induce HO-1 following LC3-II expression to protect against liver IR injury. Furthermore, phosphorylation of ERK1/2 and p38-MAPK staining increased in hemin-pretreated liver cells compared with controls, and the expression of phosphorylated ERK1/2 and p38-MAPK was down-regulated after treatment with HO-1 siRNA. We observed HO-1 expression blockade by PD98059 but not by SB203580, whereas LC3-II expression was down-regulated by both SB203580 and PD98059. Based on this and previous reports, we hypothesize that HO-1 induced autophagy may occur partly via HO-1/p38/MAPK-dependent signaling.

RIPC protects the liver by releasing biochemical messengers like free radicals to the blood, which may induce HO-1 expression or activation of nerve pathways [19]. Serum HO-1 levels were higher in the IPC group than the sham group, which were attributed to alleviating liver IRI [20]. RIPC has also been verified as a pretreatment strategy to protect liver from IRI, which is in part a benefit from the induction of HO-1 [21]. HO-1 confers cytoprotection against cell death in various models of lung and vascular injury by inhibiting apoptosis, inflammation, and cell proliferation [5]. It has been announced that the intrinsic heme-HO-1 pathway is important for both the susceptibility and severity of acute kidney injury [6]. The expression of HO-1 in mice with the *c-Jun Terminal Kinase-2* gene knocked down is significantly enhanced, which dramatically protects the mouse liver from hepatic IRI [7]. Studies have shown that hepatic HO-1 protein in rats peaks after reperfusion after rising before 6 h, and declines [22]. HO-1 increases and is induced after reperfusion, which might play a negative feedback role in the IR process. In our research, HO-1 was up-regulated in the IR group, and RIPC treatment prior to IR resulted in significant potentiated HO-1 expression, which consequently protected the liver from IRI, however, the specific mechanism of the protective role is needs further studies.

A recent study found that the regulation of autophagy via HO-1 signaling may play a protective role against liver damage [18]. The up-regulation of HO-1 in cells results from the release by injured cells, with injury achieved via ischemia of remote organs in our experiment. Cells in damage are also associated with autophagy that degrades the injured cells and organelles. Autophagy is rarely observed in normal cells without predisposing factors: intracellular (e.g., metabolic stress, aging or damaged organelles, and misfolded or aggregated proteins) and extracellular (e.g., extracellular nutrients, hypoxia-ischemia, and concentration of growth factors). The process of IR is an important factor to induce autophagy, which is confirmed by our research both in vivo and in vitro. Some have reported a protective role for autophagy in liver IRI [23], [24], and we demonstrated that the up-regulation of autophagy can protect the liver from IRI (Fig. 1). Additionally, autophagy is reported to be closely related to cell stress or cell death, and exhibits a protective role in the liver [25]–[27]. The mechanism of how autophagy limits liver cell death and alleviates IRI is unclear.

Recent evidence showed that mitophagy, the elective autophagic removal of mitochondria, plays an important role in IRI. Mitophagy, the removal of damaged mitochondria, is seen as an important mechanism. Some researchers report that damaged mitochondria are selectively removed and degraded by mitophagy. Mitochondria of some non-proliferating tissues have a life cycle in which dysfunctional mitochondria are removed by mitophagy and replaced by new mitochondria. Mitophagy plays a role in segregating and degrading dysfunctional mitochondria that might otherwise release some adverse factor such as reactive oxygen species, pro-apoptotic proteins, or other toxic mediators. Studies show that factors released from the mitochondrial intermembranous space may provide the signal that not only leads to a degradation of individual mitochondria, but also stimulate autophagy [28], [29]. We showed that cell apoptosis ratios were increased in the mineral oil treatment group and that HO-1 siRNA treatment before IR increases the rate of apoptosis compared with the IR group (P<0.05). However, there were no significant differences in rates of apoptosis between the control and HIR groups (Fig. 7A).

In our research, RIPC was used to induce autophagy, which consequently played a protective role against liver IRI. Based on our previous study, RIPC could induce HO-1 to protect against liver IRI. We found that autophagy was induced in the RIPC group in which HO-1 was also up-regulated, which consequently protected the liver from IRI. Conversely, autophagy was inhibited when HO-1 was down-regulated by Znpp, which consequently aggravated liver IRI in vivo (Fig. 3). However, no induction of HO-1 and LC3-II expression could be detected when mice underwent Znpp administration before RIPC and IR, and hepatic damage was not alleviated in the Znpp+RIPC+IR group compared with the RIPC+IR group. We also showed that autophagy was induced when HO-1 was up-regulated by hemin, which decreased biochemical enzyme levels in culture supernatants and reduced the apoptosis of liver cells. However, the inhibition of HO-1 activity decreased autophagy and induced apoptosis in cultured hepatocytes. Thus, we speculate that HO-1 is a key regulatory factor of autophagy; RIPC can also up-regulate HO-1 to induce autophagy to protect the liver from IRI.

Many studies have revealed that MAPK cascades may in part induce HO-1 mediated protection against oxidative stress and apoptosis in SH-SY5Y cells [30], [31]. It is suggested that the overexpression of HO-1 in vivo or vitro results in the activation of the ERK1/2 pathway. ERK plays an important role in the activation and translocation of Nrf2, which subsequently elevates HO-1 expression [32], [33]. In our study, we confirmed that ERK activation and subsequent activation of HO-1. In the HIR group, hemin-treated cells significantly increased ERK1/2 phosphorylation protein and HO-1 protein expression (Fig. 7B). Similarly, we found a slight reduction of ERK1/2 phosphorylation in the HSIR group. We concluded that ERK may be more sensitive to the pre-signal of increasing HO-1. Subsequently, we found that PD98059 could decrease HO-1 expression, suggesting that ERK1/2 may be an upstream activator of HO-1.

Some researchers hypothesize that protein catabolism, intracellular stresses, and autophagy may be cellular responses to increased levels of intracellular heme. The upregulation of cellular HO-1 results from the release of heme from injured cells, which was achieved via ischemia of remote organs in our experiments and is also associated with autophagy that degrades injured cells and organelles, such as damaged and dysfunctional mitochondria, and then inhibits cell apoptosis. The most carbon monoxide (CO) produced ex vivo is from heme degradation, and CO may act as an important anti-inflammatory and cytoprotective method to defend against liver IRI [34]. Some studies have found that the mitochondrial ROS induced by CO could increase the phosphorylation of p38 MAPK to induce autophagy; our study also revealed a positive correlation between HO-1, p38 phosphorylation, and LC3-II. Given that an increase of phosphorylated p38-MAPK was observed in this process, it seems that the p38-MAPK pathway might be activated by the process [35]. LC3-II expression was down-regulated by SB203580 (Fig. 7), suggesting that p38 MAPK may be an upstream activator of autophagy. HO-1 may increase the phosphorylation of p38-MAPK to induce autophagy and reduce cell apoptosis; however, the inhibition of HO-1 could decrease the phosphorylation of p38-MAPK and weaken autophagy, thus increasing cell apoptosis.

Activated ERK1/2, which may be induced via increased HO-1, can strengthen the expression of HO-1 feedback. In this study, we report that the induction of HO-1 may partly activate the expression of autophagy via the p38-MAPK pathway, and the adaptive response to stress (autophagy), may result in the slower accumulation of damaged and dysfunctional mitochondria, which is anti-apoptotic. In conclusion, RIPC induced the up-regulation of HO-1, which may act as a key waypoint in autophagy and apoptosis, triggered a signal pathway of p38-MAPK to induce autophagy, and then devour the damaged mitochondria to inhibit apoptosis, and eventually protected hepatic cells from IRI.
